# A tool for incorporating interprofessional perspectives into dental students decision‐making: A 2‐year follow‐up on this learning outcome

**DOI:** 10.1002/cre2.615

**Published:** 2022-06-19

**Authors:** Trevor W. Craig, Carissa L. Comnick, Kecia S. Leary, Jennifer E. Hartshorn, David C. Johnsen, Leonardo Marchini

**Affiliations:** ^1^ D4 Predoctoral Dental Student University of Iowa College of Dentistry Iowa City Iowa USA; ^2^ Division of Biostatistics University of Iowa College of Dentistry Iowa City Iowa USA; ^3^ Department of Pediatric Dentistry University of Iowa College of Dentistry Iowa City Iowa USA; ^4^ Department of Preventive and Community Dentistry University of Iowa College of Dentistry Iowa City Iowa USA

**Keywords:** dental education, geriatric dentistry, interprofessional education

## Abstract

**Objectives:**

To analyze student performance when using a sustainable teaching tool developed to guide learning toward interprofessional perspectives.

**Methods:**

This study compiled data about D4 students’ performance when using an interprofessional education (IPE) teaching tool reported previously in this journal, during their 5‐week Geriatric and Special Needs Program rotation in the academic years 2018–2019 and 2019–2020. Ninety‐two students were introduced to IPE concepts and teaching tools during their orientation. Students were then asked a question regarding the perspective of each healthcare team member and whether they would contact these healthcare team members for collaboration during the provision of oral care with regard to various patient cases. Students were scored on whether they answered the question about the perspective of each health care team member. The same two independent evaluators also noted whether the student thought each member of the health care team should be contacted.

**Results:**

A majority (90.2%–95.7%) of dental students applied their knowledge to questions regarding each health care team member's perspectives. The profession that dental students most often indicated they wished to contact for collaboration was primary care providers (*n* = 70; 76.1%), followed by family caregivers (*n* = 54; 58.7%), and pharmacists (*n* = 46; 50.0%). The results of the interrater agreement between the two‐faculty scoring students were between 86.7% and 100%.

**Conclusions:**

The teaching tool is sustainable and succinct. Students considered the perspectives of each health care team member at a rate above 90%, and the interrater agreement was high among the faculty evaluators. Students considered contacting primary care providers, family caregivers, and pharmacists more often than the other health care team members. We see this model as one approach to begin the articulation of learning outcomes for IPP.

## 1 INTRODUCTION

Interprofessional Education and Practice (IPE/IPP) have been endorsed by every major health group to be a key to improved chances for favorable patient outcomes (Brandt et al., 2014; D'Amour et al., 2005; Gauger et al., 2018; Haresaku et al., 2021; Harnagea et al., 2017; Interprofessional_Education_Collaborative 2016; O'Malley & Reschovsky, 2011). To accomplish IPP, it is compelling for each member of the health care team to consider and even incorporate the key questions from every other team member for the next patient. In a previous paper in this journal, the authors offered a model for a learning outcome for Interprofessional (or Collaborative) Practice (Leary et al., 2019). The purpose of this paper is to consolidate the learning model from the previous paper and to assess its sustainability. To build on the previous work, some repetitions from the previous paper are incorporated into this paper and acknowledged. The current work incorporates the input from the interprofessional team as a first step to developing a learning outcome with the promise to improve patient outcomes.

Much progress has been made to enhance the culture of IPP. Extensive national efforts have been made to get health care providers together to coordinate care better so that outcomes are improved. Several gaps remain in realizing a true IPP. One gap is the scarcity of a model or learning guide for each team member to incorporate the thinking of each other team member. In addition, learning outcomes and learning guides have been elusive with a “wholesale lack of consistency in defining and describing learning [performance] outcomes and their assessment” for IPP, and “the continual lack of longitudinal studies remains problematic” (Thistlethwaite et al., 2010). One dilemma is the distinction between competencies and learning outcomes. Definitions of competency have centered around capability, capacity, and competence. These perspectives are essential for gaining a larger view of capability, but they fall short of articulating what the practitioner is to *do* when interacting with the next patient. In the emulation model, the thought process of the expert is the outcome, the learning guide, and the assessment instrument and captures what the student is to *do* in assessing the next patient. A goal is to develop an outcome to guide learning and assess the performance of the student in the *act* of critical thinking. A task for the educator is to create or derive the act of critical thinking and inspire students to adopt this approach. An example of a learning outcome in critical thinking is a thought process of the expert emulating the intended activity (Johnsen, 2013; Johnsen et al., 2012; Lane & Stone, 2006). An assumption is that to effectively guide learning and assess performance, the first step is to know what the student is to do. Without knowing what the student is to do, the question arises of whether meaningful guidance and performance assessment can happen. A challenge in IPP is that there are multiple experts and no single expert.

The general purpose of this project is to follow up on the effectiveness of an emulation model for students in the act of critical thinking to apply key questions derived from multidisciplinary team members to the next patient (Table 1). The model was implemented 3 years ago (with 2 years of results) as an introductory IPP exercise during a student evaluation of a complex dental patient. The exercise was incorporated into a larger exercise in risk assessment in a geriatrics and special needs clinic (Leary et al., 2019; Marchini et al., 2017). This exercise introduced a succinct summary of the “first questions” each team member considered important during patient evaluations. The first purpose is to test the sustainability of that model. Since the paper appeared introducing the model, we are not aware of another peer‐reviewed model to focus on explicit learning/performance outcome(s) for IPP. A secondary purpose is to explore the students’ propensity for engaging fellow team members with their patients.

**Table 1:** Interprofessional practice skillset (Leary et al., 2019)

Provider question to ask:
*Patient*: Preferences and expectations? *Primary care*: Prioritization of condition(s) life threatening or affecting health? *Pharmacy*: Patient problems that are (or are potentially) drug related? *Nursing*: Patient capacity to subscribe to treatment recommendations? *Dentistry*: Dental conditions/risk factors that affect (or are indicators of) general health?; preventive measures for oral health based on overall health? *Nutrition*: Nutritional factors contributing to disease/condition, asking what the patient eats and drinks on a daily basis? *Social worker*: Barriers/solutions for care based on the home situation (money, transportation, organization, availability of assistance, consent, etc.)? *Physical therapist/occupational therapist*: Long‐term outcomes for therapy programs? *Family caregiver*: Person(s) responsible for the patient's daily living activities (consent, finances, etc.)? *Patient*: Assent?

Another gap in developing a learning model for IPP is the lack of any baseline for the level of awareness each health care team member has about the thinking of other team members. A second purpose is, therefore, to report 2 years of results from 3 years of model implementation to systematically include the primary question recommended for the next patient by other members of the team. In other words, did students systematically ask the questions recommended by fellow team members regarding their patients, and how frequently did dental students feel it important to seek input from another team member? While the project is for dental students, the approach could be used for any health discipline. While it is beyond the scope of this project to assess patient outcomes, it seems logical that a first step would be to follow the recommendations of experienced health care team members in patient assessment. We are not aware of this approach being used previously.

Concepts for the IPP model are based on emulating the thought processes of the experts on the health care team (Leary et al., 2019). Previously reported critical thinking emulations have taken the thought process of people from a single discipline, for example, treatment planning, risk assessment, and so on (Benner, 1982; Guzman‐Armstrong et al., 2014; Johnsen, 2013; Johnsen et al., 2009, 2020; Leary et al., 2019; Lane & Stone, 2006; Marshall et al., 2011, 2017; Marchini et al., 2017). The goal is to derive the expert's thought process succinctly enough for the novice to apply to the next patient or situation. The thought process becomes the learning outcome, the learning guide, and the assessment instrument (Johnsen, 2013; Johnsen et al., 2012; Lane & Stone, 2006). The IPP model derives the central thought process of each member of the health care team. The thought process for each team member is derived by asking, “What is the first question you want every member of the health care team to ask when they see their next patient?” The learning outcome for IPP is thus the collective thought processes collating the “first questions” for each member of the health care team (Table 1). While the questions are derived from respective health care team members, the questions are really common sense. We are not aware of this approach being applied previously.

The collection of thought processes of individual health care team members—the patient, primary care, pharmacy, nursing, social work, nutrition, dentistry, physical therapy, family caregiver, ending with the patient—had not been previously reported. The patient is considered part of the health care team.

## 2 AIMS

The first purpose is to test the sustainability of an emulation model by asking the first question of the primary care provider, pharmacist, nurse, dentist, nutritionist, physical therapist, social worker, and family caregiver. Since the paper appeared introducing the model, we are not aware of another peer‐reviewed model to focus on explicit learning/performance outcome(s) for IPP. A secondary purpose is to explore the students’ propensity for engaging fellow team members with their patients.

## 3 METHODS

This study was approved by the Institutional Review Board of the University of Iowa, Iowa City, Iowa, USA, as an exempt project in 2015 (IRB201512721). This study compiled data about D4 students’ performance using an interprofessional education (IPE) teaching tool, during their 5‐week Geriatric and Special Needs Program (*GSNP*) rotation in the academic years 2018–2019 and 2019–2020 (Table 1). During their previous dental coursework, the students have been exposed to the content related to IPP in different courses, such as oral surgery, pharmacology, pediatric dentistry, and others. Students also participated in IPE exercises with other health professional students in University‐wide IPE exercises. During their orientation to the GSNP rotation, students reviewed basic IPE concepts and were introduced to specific IPE teaching tools. Students were then guided to (1) systematically ask the questions that each health care team member recommended for each patient and (2) whether they would contact these health care team members for collaboration during the provision of oral care. In the present project, students recommended contacting a specific member of the health team. There was no follow‐up as to whether the identified member of the health care team was actually contacted.

Students were scored by two independent evaluators on whether they answered the question about the perspective of each health care team member as either applied the step (A), missed the step (M), or marked it as not applicable (N/A) to their patient/case. The same two evaluators also noted whether the student thought each member of the health care team should be contacted. The questions on who to consult elicited intuitive responses. The next step is to add structure to the discussion on who to consult and why. The same is true for the student intuitively designating a question as “Not Applicable.”

These data reflect evaluations completed in three time periods between May 2018 and March 2020, and responses are split into three groups by time. Group 1 spans May 16, 2018, to December 12, 2018, group 2 spans February 6, 2019, to August 1, 2019, and group 3 spans September 11, 2019, to March 11, 2020.

Interrater agreement between the two raters was assessed via Cohen's *κ* and percent agreement, calculated within each health care team member's question and combining responses from all three time periods. Because the interrater agreement was high across all categories, the first rater's answer for each question was used when possible, while the other rater's answer was only used if the first rater's answer was missing. These final data were tabulated overall and by time period. To examine differences in rates of different answers across the three groups, *χ* ^2^ and Fisher's exact tests were used. All analyses were performed in R version 4.0.0 using a 5% significance level. We did not adjust for multiple comparisons.

The didactic/clinical thread before implementation of the IPP skillset included:

- For primary care: Physical assessment as part of the Oral Surgery rotation and health histories of every patient
- Pharmacology and Pharmacy courses and ongoing clinical consults
- Nutrition fundamentals
- Social work skillset in the 5‐week Geriatrics rotation and social work concepts in Quality Assurance assessments with patients.

There were no explicit didactic courses in nursing or physical therapy.

## 4 RESULTS

A high percentage of students asked and responded to the provider question for each discipline. Results are in Table 2. For respective discipline categories, over 90% of students (ranging from *n* = 88 to *n* = 83 depending on the question) asked and responded to the question for each discipline. A lower percentage of students recommended contacting any of the disciplines than asked and responded to the provider question from each discipline. Results are in Table 3. The lowest percentage for asking and responding to a question from a discipline was 90.2% for “Nutrition” (*n* = 83), “Social Work” (*n* = 83), and “occupational therapy/phylical therapy” (*n* = 83). The discipline that dental students most often indicated they wished to contact for collaboration was primary care providers (*n* = 70; 76.1%), followed by family caregivers (*n* = 54; 58.7%), and pharmacists (*n* = 46; 50.0%). Less commonly indicated to be contacted were nurses (*n* = 30; 32.6%), nutritionists (*n *= 30; 32.6%), and physical therapists/occupational therapists (*n* = 22; 23.9%). The results of the interrater agreement between the two‐faculty scoring students were between 86.7% and 100%. Students who were judged to “Apply” a step were almost always judged to “Grasp” the meaning of the step. Although there are no data to show student performance in systematically asking the questions of each discipline before the IPP exercise was introduced, faculty agree that students did not explicitly ask discipline‐based questions before the learning exercise was introduced. The question, “Do you recommend contacting a team member about this patient?” was also significantly different in the family caregiver—groups 2 and 3 had higher rates of contacting the family caregiver than group 1 (*p* < .001). No other significant differences were seen.

**Table 2:** Group analysis; “Did the student ask and respond to the question from each discipline, “What is the first thing every member of the team should ask about the next patient?.”

	All (*N* = 92)	Group 1 (*N* = 27)	Group 2 (*N* = 33)	Group 3 (*N* = 32)	*p* Value
Answer primary care provider					.617
A	87 (94.6%)	26 (96.3%)	30 (90.9%)	31 (96.9%)	
M	5 (5.43%)	1 (3.70%)	3 (9.09%)	1 (3.12%)	
Answer pharmacist					1.000
A	86 (93.5%)	26 (96.3%)	30 (90.9%)	30 (93.8%)	
M	4 (4.35%)	1 (3.70%)	2 (6.06%)	1 (3.12%)	
N or N/A	2 (2.17%)	0 (0.00%)	1 (3.03%)	1 (3.12%)	
Answer nurse					.680
A	87 (94.6%)	25 (92.6%)	31 (93.9%)	31 (96.9%)	
M	4 (4.35%)	1 (3.70%)	2 (6.06%)	1 (3.12%)	
N or N/A	1 (1.09%)	1 (3.70%)	0 (0.00%)	0 (0.00%)	
Answer dentist/DH					1.000
A	85 (92.4%)	25 (92.6%)	30 (90.9%)	30 (93.8%)	
M	4 (4.35%)	1 (3.70%)	2 (6.06%)	1 (3.12%)	
N or N/A	3 (3.26%)	1 (3.70%)	1 (3.03%)	1 (3.12%)	
Answer nutritionist					.914
A	83 (90.2%)	24 (88.9%)	30 (90.9%)	29 (90.6%)	
M	5 (5.43%)	2 (7.41%)	2 (6.06%)	1 (3.12%)	
N or N/A	4 (4.35%)	1 (3.70%)	1 (3.03%)	2 (6.25%)	
Answer PT/OT					.793
A	83 (90.2%)	25 (92.6%)	30 (90.9%)	28 (87.5%)	
M	4 (4.35%)	1 (3.70%)	2 (6.06%)	1 (3.12%)	
N or N/A	5 (5.43%)	1 (3.70%)	1 (3.03%)	3 (9.38%)	
Answer social worker					.644
A	83 (90.2%)	26 (96.3%)	29 (87.9%)	28 (87.5%)	
M	4 (4.35%)	1 (3.70%)	2 (6.06%)	1 (3.12%)	
N or N/A	5 (5.43%)	0 (0.00%)	2 (6.06%)	3 (9.38%)	
Answer family caregiver					.431
A	88 (95.7%)	26 (96.3%)	31 (93.9%)	31 (96.9%)	
M	3 (3.26%)	0 (0.00%)	2 (6.06%)	1 (3.12%)	
N or N/A	1 (1.09%)	1 (3.70%)	0 (0.00%)	0 (0.00%)	

Abbreviations: DH, dental hygienist; OT, occupational therapy; PT, phylical therapy.

**Table 3:** Group analysis (continued) “Do you recommend contacting team members about this patient?.”

	All (*N* = 92)	Group 1 (*N* = 27)	Group 2 (*N* = 33)	Group 3 (*N* = 32)	*p* Value
Contacted primary care provider					.935
Y	70 (76.1%)	20 (74.1%)	25 (75.8%)	25 (78.1%)	
N or N/A	22 (23.9%)	7 (25.9%)	8 (24.2%)	7 (21.9%)	
Contacted pharmacist					.649
Y	46 (50.0%)	12 (44.4%)	16 (48.5%)	18 (56.2%)	
N or N/A	46 (50.0%)	15 (55.6%)	17 (51.5%)	14 (43.8%)	
Contacted nurse					.051
Y	30 (32.6%)	6 (22.2%)	16 (48.5%)	8 (25.0%)	
N or N/A	62 (67.4%)	21 (77.8%)	17 (51.5%)	24 (75.0%)	
Contacted dentist/DH					.909
Y	44 (47.8%)	12 (44.4%)	16 (48.5%)	16 (50.0%)	
N or N/A	48 (52.2%)	15 (55.6%)	17 (51.5%)	16 (50.0%)	
Contacted nutritionist					.322
Y	30 (32.6%)	6 (22.2%)	11 (33.3%)	13 (40.6%)	
N or N/A	62 (67.4%)	21 (77.8%)	22 (66.7%)	19 (59.4%)	
Contacted PT/OT					.935
Y	22 (23.9%)	7 (25.9%)	8 (24.2%)	7 (21.9%)	
N or N/A	70 (76.1%)	20 (74.1%)	25 (75.8%)	25 (78.1%)	
Contacted social worker					.113
Y	41 (44.6%)	10 (37.0%)	12 (36.4%)	19 (59.4%)	
N or N/A	51 (55.4%)	17 (63.0%)	21 (63.6%)	13 (40.6%)	
Contacted family caregiver					<.001
Y	54 (58.7%)	10 (37.0%)	28 (84.8%)	16 (50.0%)	
N or N/A	38 (41.3%)	17 (63.0%)	5 (15.2%)	16 (50.0%)	

Abbreviations: DH, dental hygienist; OT, occupational therapy; PT, phylical therapy.

## 5 DISCUSSION

This Emulation Model for IPP is shown as sustainable in eliciting dental students to systematically ask and respond to questions deemed important for every member of the health care team to ask regarding the next patient. The collective thought processes of the team members became the learning outcome, learning guide, and assessment instrument. The high‐performance rate (over 90% for students asking and responding to the central question for all disciplines) is interpreted to mean that the exercise was effective in engaging students in incorporating the initial thinking of each discipline. The lower percentages of students who would recommend contacting respective disciplines is more difficult to interpret. One explanation could be that the student had enough confidence to manage the patient without actual consultation. A future direction in refining the exercise will be to add the question, “Why would you contact team member X?” Some association is seen between exposure to explicit clinical or clinically oriented material (whether didactic or in‐clinic) and articulation of that discipline in the IPP exercise. The inclusion of “primary care provider” was most articulated by students and is the area with more extensive didactic and clinical exposure. Pharmacy is next most common in the IPP exercise and also has extensive didactic and clinical exposure. Nursing is less frequently cited and is associated with no explicit didactic content in Nursing. Similarly, there is no explicit instruction in Physical Therapy in the dental curriculum. Nutrition has an extensive didactic component, but not a clinical rubric. Social work has little explicit didactic coursework, but there is an extensive quality assurance component with elements of social work. Yet to be determined is how effectively the students will be in identifying explicit patent problems once the question is asked. Also, yet to be determined is the effectiveness of the student in recommending treatments for the patient once the question is asked and responses are obtained.

Having established sustainability and practical application for the emulation model for IPP, the next step is to articulate how effective students could be in improving patient acceptance of recommended treatment using this model. It is likely that a separate critical thinking skill will be needed to guide learning on patient adherence to recommended treatment.

While beyond the scope of this project, the next step would be to find the impact on patient outcomes (Glick et al., 2016). The theory is that practitioners systematically asking a question will get better at answering it over time. The authors submit that systematically asking this battery of questions improves the likelihood of improved pursuit of patient issues, both problem‐related and treatment‐related. It is logical that not asking the questions of each discipline leaves gaps in patient care.

## 6 LIMITATIONS

Generalizations of the conclusions to other health disciplines have limitations, yet the conclusions are mostly common sense regarding other disciplines and would seem to have some generalizability.

## 7 CONCLUSIONS

After 2 years, the critical thinking model for IPP is seen to be sustainable and succinct. In this 2‐year observation period, students considered the perspectives of each health care team member at a rate above 90%, and the interrater agreement was high among the faculty evaluators. While we do not expect for students to have an in‐depth grasp for the thinking of an experienced health team member from another discipline, results are seen as a first step in at least considering the first priority question for each team member. We are not aware this step has been previously taken. For student patient/cases in the Geriatrics and Special Needs Clinic, students considered contacting primary care providers, family caregivers, and pharmacists more often than the other health care team members. Since the initial reporting of implementing this model, we are not aware of other models emerging with explicit learning outcomes for IPP.

## AUTHOR CONTRIBUTIONS

**Trevor W. Craig**: Participation in designing the project, collating raw data, reviewing data, and designing and reviewing the manuscript. **Carissa L. Comnick**: Participation in designing the project, reviewing data, conducting statistical tests, designing tables, and designing and reviewing the manuscript. **Kecia S. Leary**: Participation in designing the project, in reviewing data, and designing and reviewing the manuscript. **Jennifer E. Hartshorn**: Participation in designing the project, conducting seminars where data were gathered, reviewing data, and designing and reviewing the manuscript. **David C. Johnsen**: Participation in designing the project, reviewing data, and designing and reviewing the manuscript. **Leonardo Marchini**: Participation in designing the project, conducting seminars where data were collected, reviewing data, and designing and reviewing the manuscript.

## CONFLICT OF INTEREST

The authors declare no conflict of interest.

## Data Availability

Data is openly available in a public repository that issues datasets with DOIs Comments to Payment Admin
